# Shades of Blue: A Case Series of Acquired Methemoglobinemia

**DOI:** 10.7759/cureus.58312

**Published:** 2024-04-15

**Authors:** Bharatsing D Rathod, Nilesh Kamble, Onkar Awadhiya, Udit Narang, Rajashree S Khot, Sunita Kumbhalkar

**Affiliations:** 1 General Medicine, All India Institute of Medical Sciences, Nagpur, IND

**Keywords:** chocolate brown-colored blood, cyanosis, methylene blue, saturation gap, methemoglobinemia

## Abstract

Acquired methemoglobinemia (MetHb) is a rare but potentially life-threatening condition that has varied etiology, usually toxin- or drug-induced. We had five cases of acquired methemoglobinemia during six months. Their presentation varied from an asymptomatic state to respiratory distress. The presence of cyanosis and low oxygen saturation (SpO_2_), despite normal partial pressure of oxygen (PaO_2_) and chocolate brown-colored blood, were diagnostic clues present in all cases. A high level of methemoglobinemia was detected on arterial blood gas (ABG), confirming the diagnosis. Methylene blue was used as an antidote along with supportive care in symptomatic cases. All these cases of methemoglobinemia recovered completely. A high index of suspicion for methemoglobinemia should be maintained in cases presenting with persistent hypoxia or cyanosis despite normal PaO_2_.

## Introduction

Acquired methemoglobinemia (MetHb) is an uncommon but potentially life-threatening condition [1]. Methemoglobinemia causes a diminution of the oxygen-carrying capacity of circulating hemoglobin due to the conversion of some or all of the four iron species from the reduced ferrous (Fe2+) state to the oxidized ferric (Fe3+) state. The inability of ferric iron to bind and transport oxygen results in functional anemia [2]. Acquired methemoglobinemia has varied etiology, including some agrochemical compounds (e.g., herbicides and fertilizers) and drugs such as nitrates, antimalarials, and topical anesthetics. Exposure to these substances causes the oxidation of the hemoglobin either directly or indirectly, resulting in the production of methemoglobin. The clinical presentation of acquired methemoglobinemia varies from an asymptomatic state to life-threatening respiratory distress. Here, we present five cases of acquired methemoglobinemia that came to us within six months.

## Case presentation

Case 1

A 19-year-old male with a case of acute undifferentiated fever and no previous comorbidities was referred from a private hospital due to decreased oxygen saturation (SpO_2_). He had had a fever with chills for four days. He had received empirical antimalarial therapy (i.e., chloroquine and primaquine) for three days. He developed cyanosis and hypoxia, so he was referred for further management. He was asymptomatic and hemodynamically stable but had central cyanosis and SpO_2_ of 88% at room air. There was no dyspnea or tachypnea. Systemic examination was normal. His arterial blood color was chocolate brown. His arterial blood gas (ABG) report showed a pH of 7.36, partial pressure of oxygen (PaO_2_)_ _of 108 mmHg, and partial pressure of carbon dioxide (pCO_2_) of 39.8 mmHg. His methemoglobin level was 16.6%. His chest X-ray was normal. His complete blood count, kidney function test, and liver function tests were within normal limits. The peripheral smear for malarial parasites was negative, and rapid tests for malaria antigen (histidine-rich protein {HRP2}) were also negative. The diagnosis of drug-induced methemoglobinemia was maintained, and he was observed. He remained asymptomatic after 72 hours of observation. His cyanosis disappeared, and the repeat ABG report on day 3 showed a methemoglobin level of 1.6%.

Case 2

A 72-year-old male farmer presented with complaints of breathlessness, chest pain, vomiting, hiccups, and generalized weakness for five days. He had been diagnosed with systemic hypertension and type 2 diabetes mellitus two years ago and had been taking amlodipine (5 mg) and metformin (500 mg) once per day. He had a history of relapsed multibacillary Hansen’s disease with type 2 lepra reaction, for which he had been started on clofazimine and prednisolone for the last 15 days. His vitals on admission were as follows: afebrile, pulse rate (PR) of 96/minute, regular; blood pressure (BP) of 140/80 mmHg; and respiratory rate (RR) of 26/minute. General examination revealed cyanosis with SpO_2_ of 85% at room air; thickened bilateral ulnar, common peroneal, and greater auricular nerves; wasting of the thenar and hypothenar muscles of the hands; and circinate healed post-inflammatory hyperpigmentation present over the trunk. Systemic examination was normal. ABG analysis revealed partially compensated acute respiratory alkalosis (pH, 7.533; pCO_2_, 19.4 mmHg; PaO_2_, 204 mmHg; bicarbonate {HCO_3_}, 15.9 mmol/L; and arterial oxygen saturation {SaO_2_}, 99%) with methemoglobin levels of 12%. The repeat ABG after two hours revealed a rising trend of MetHb (21%) with worsening cyanosis, and his SpO_2_ decreased further to 78%.

We made the diagnosis of clofazimine-induced methemoglobinemia in a case of systemic hypertension with type 2 diabetes mellitus with relapsed multibacillary leprosy and type 2 lepra reaction. Given his symptomatic status, he was treated with methylene blue along with the prompt discontinuation of clofazimine.

Case 3

A 56-year-old male with a diagnosed case of rheumatoid arthritis had been on a regular treatment of methotrexate, hydroxychloroquine, and folic acid for the last five years. He presented with complaints of weakness and breathlessness for five days. He had stopped taking disease-modifying antirheumatic drugs (DMARDs) and had taken some herbal medications in powder form, details of which were not available. His vitals were normal except for low SpO_2_. He was having central cyanosis, and his SpO_2_ was 82%. The ABG revealed a pH of 7.32 mmHg, pCO_2_ of 35 mmHg, and PaO_2_ of 112 mmHg. His arterial blood color was dark brown, and his methemoglobin level was 36%. He was treated with methylene blue and supportive care.

Case 4

A 26-year-old male farmer presented with acute consumption of a “grace compound” containing botanical oil (25% w/v), followed by two episodes of vomiting and breathlessness. He was afebrile with a PR of 98/minute, tachypneic with an RR of 26/minute, and normotensive. There was no particular smell of insecticide compound on him. He presented with central cyanosis and low saturation on pulse oximetry (78%). His systemic examination was normal. He was started on high-flow oxygen therapy by mask, but his saturation did not improve. ABG analysis revealed a pH of 7.32 mmHg, pCO_2_ of 29 mmHg, and PaO_2_ of 105 mmHg. The clinical suspicion of methemoglobinemia arose due to cyanosis, low saturation despite oxygen therapy, and the presence of a typical “saturation gap,” that is, a low saturation on pulse oximetry (78%) and high PaO_2_ (105 mmHg) on ABG analysis and the presence of chocolate-colored arterial blood. His initial methemoglobin level was 41%. He was treated with methylene blue along with supportive care. A repeat dose of methylene blue was administered after 60 minutes due to persistent cyanosis and breathlessness. He responded well to treatment.

Case 5

A 32-year-old male recently diagnosed with congenital cyanotic heart disease (bicuspid aortic valve) with tuberculoid leprosy was started on anti-Hansen’s disease treatment (rifampicin and dapsone). He developed fatigue, headache, arthralgia, and breathlessness 15 days after starting anti-Hansen’s disease therapy. He presented with central cyanosis and hypoxia on admission. His SpO_2_ was 80%, and PaO_2_ was 128 mmHg. His blood was dark brown, and his methemoglobin level was 38%. The diagnosis of drug-induced (dapsone) methemoglobinemia was maintained, and his anti-Hansen’s disease medications were stopped. He was treated with methylene blue.

The clinical and laboratory features of all five cases are summarized in the Appendices.

All five cases had normal glucose-6-phosphate dehydrogenase (G6PD) levels. All cases, except case 1, were treated with methylene blue, and they all recovered from methemoglobinemia completely.

## Discussion

In the present case series, we encountered five cases of methemoglobinemia caused by medication and toxins. Belzer and Krasowski, in their retrospective study at the University of Iowa Hospitals and Clinics (UIHC), analyzed causes of methemoglobinemia across approximately 27 years (from May 2009 to June 2023). They did not encounter any congenital causes of methemoglobinemia. Dapsone was the most common cause of methemoglobinemia, followed by nitrate compounds. They noticed that fatigue, cyanosis, and respiratory difficulty were common symptoms in cases with more than 10% methemoglobin level [3].

The ferric state of heme in methemoglobinemia interferes with tissue oxygen delivery [2]. High methemoglobin levels can cause hypoxic tissue damage, systemic inflammation, cyanosis, and chocolate-colored blood.

Methemoglobinemia is either congenital or acquired. Acquired methemoglobinemia is more common and most often caused by exposure to exogenous oxidizing substances such as some agrochemical compounds and medications such as antimalarials, anesthetic drugs, and nitrates [4,5]. The normal level of methemoglobin is 1.0%-1.5%. Methemoglobinemia is diagnosed when levels rise above 5%. The clinical presentation of methemoglobinemia is varied and depends upon the level of methemoglobin (%), the patient’s cardiovascular reserve, and the usual hemoglobin level. Symptoms develop typically at levels greater than 10%-20% of total hemoglobin, and levels greater than 30%-40% are considered life-threatening [2]. Patients with an elevated methemoglobin concentration may initially develop relatively mild symptoms such as dyspnea, headache, lethargy, and fatigue. However, at higher methemoglobin levels, symptoms may progress to respiratory distress, altered mentation, seizure, profound cyanosis, dysrhythmias, and death [2,6]. When methemoglobin levels rise above 20%, the blood develops a chocolate brown color [7,8]. Clofazimine-induced methemoglobinemia has rarely been reported in the literature. Methemoglobinemia is suspected based on clinical findings, that is, generalized cyanosis not correlating with respiratory status, low SpO_2_ with normal PaO_2_, and chocolate brown-colored blood [4]. A SaO_2_-SpO_2_ gap of more than 5% should also raise suspicion of methemoglobinemia [9]. Traditional dual wavelength pulse oximetry is inaccurate in measuring oxygen saturation in the setting of methemoglobinemia. It usually reads 85% saturation irrespective of actual oxygenation status because methemoglobinemia interferes with the accuracy of pulse oximetry by causing modified light absorption patterns [10]. Co-oximetry is therefore advocated for measuring methemoglobin levels. Newer ABG machines can directly measure methemoglobin.

Acute toxic methemoglobinemia with methemoglobin levels above 30% (or lower if symptomatic from hypoxia) is a medical emergency. Treatment includes the prompt discontinuation of the offending agents or medication; the institution of appropriate supportive care including intravenous (IV) access, hydration for hypotension, ventilator support for respiratory compromise, or treatments targeted to neurologic complications (antiepileptics); and the administration of the antidote methylene blue at a dose of 1 mg/kg IV bolus. A repeat dose of methylene blue after one hour may be given according to the patient’s methemoglobin level [11]. Methylene blue reacts within red blood cells (RBC) to form leuco-methylene blue, an oxidized hemoglobin-reducing agent, converting the ferric iron to a ferrous state. Methylene blue is indicated in symptomatic methemoglobinemia regardless of methemoglobin level and/or patients with methemoglobin levels over 30%. Asymptomatic patients with methemoglobin levels of less than 30% should be closely monitored for the development of any symptoms. Methylene blue is not used in individuals with G6PD deficiency as it can cause hemolytic anemia. High-dose ascorbic acid (1.5-3 g IV every six hours) may be tried in such individuals [12].

## Conclusions

Acquired methemoglobinemia is a rare but treatable condition that causes significant morbidity and even mortality. It is usually caused by exposure to certain drugs and compounds. The severity of symptoms depends upon the percentage of methemoglobin in the blood. A heightened index of suspicion must be maintained in cases of hypoxia or cyanosis refractory to supplemental oxygen use and the presence of a typical “saturation gap” (i.e., low saturation on pulse oximetry, high PaO_2_ on ABG analysis, and the presence of chocolate-colored blood). Treatment depends upon symptom status and the level of methemoglobin. Emergency services should be equipped with facilities to detect and treat methemoglobinemia.

## Appendices

Table 1 shows the clinical and laboratory features of all five cases.

**Table 1:** Clinical and laboratory features of cases of methemoglobinemia HTN, hypertension; T2DM, type 2 diabetes mellitus; MetHb, methemoglobinemia; SpO_2_, oxygen saturation; PaO_2_, partial pressure of oxygen

Clinical and laboratory parameter	Case 1	Case 2	Case 3	Case 4	Case 5
Age (years)	19	72	56	26	32
Gender	Male	Male	Male	Male	Male
Symptoms	Asymptomatic	Breathlessness, chest pain, vomiting, hiccups, and generalized weakness	Fatigue and breathlessness	Vomiting and breathlessness	Fatigue, headache, arthralgia, and breathlessness
Comorbidities/illnesses	Malarial fever	Systemic HTN, T2DM, and multibacillary leprosy with type 2 lepra reaction	Rheumatoid arthritis	Nil	Paucibacillary leprosy
Peak MetHb level (%) (normal range: 0%-2%)	16.6	21	36	41	38
SpO_2_ (%) (normal range: 95%-100%)	88	78	82	78	80
PaO_2_ (mmHg) (normal range: 75-100 mmHg)	108	204	112	105	128
Causative agent	Antimalarial drug (primaquine)	Clofazimine	Herbal medications	“Grace compound” containing botanical oil (25% w/v)	Dapsone
Treatment	None	Methylene blue	Methylene blue	Methylene blue	Methylene blue
Outcome	Recovered	Recovered	Recovered	Recovered	Recovered
